# The critical role of Gαi3 in oral squamous cell carcinoma cell growth

**DOI:** 10.1038/s41420-024-02191-0

**Published:** 2024-10-01

**Authors:** Quan Li, Zhiyue Huang, Zihan Li, Jianlin Fan, Ke Li

**Affiliations:** 1 Department of Stomatology, The Second Affiliated Hospital of Soochow University, Suzhou, China; 2 Department of Burn and Plastic Surgery, The First Affiliated Hospital of Soochow University, Suzhou, China

**Keywords:** Targeted therapies, Squamous cell carcinoma

## Abstract

The identification of novel and effective therapeutic targets for oral squamous cell carcinoma (OSCC) is of paramount importance. This study investigates the expression, potential functions, and mechanistic insights of G protein inhibitory subunit 3 (Gαi3) in OSCC. Gαi3 is found to be upregulated in human OSCC tissues as well as in various primary and established OSCC cells. In different OSCC cells, silencing of Gαi3 through shRNA resulted in inhibited cell proliferation and migration, while also inducing apoptosis. Knockout (KO) of Gαi3 via the CRISPR/Cas9 method produced significant anti-cancer effects in OSCC cells. Conversely, ectopic overexpression of Gαi3 enhanced OSCC cell growth, promoting cell proliferation and migration. Gαi3 plays a crucial role in activating the Akt-mTOR signaling pathway in OSCC cells. Silencing or KO of Gαi3 led to decreased phosphorylation levels of Akt and S6K, whereas overexpression of Gαi3 increased their phosphorylation. Restoration of Akt-mTOR activation through a constitutively active mutant Akt1 mitigated the anti-OSCC effects induced by Gαi3 shRNA. In vivo, Gαi3 silencing significantly suppressed the growth of subcutaneous OSCC xenografts in nude mice, concomitant with inactivation of the Akt-mTOR pathway and induction of apoptosis. Collectively, these findings underscore the critical role of Gαi3 in OSCC cell growth both in vitro and in vivo.

## Introduction

Oral squamous cell carcinoma (OSCC), representing 95% of all forms of head and neck cancer, presents a significant global health challenge due to its high morbidity and mortality rates, with a 5-year survival rate stagnating around 50% [1–3]. Despite the critical importance of early diagnosis and treatment, traditional therapeutic modalities—surgery, radiotherapy, and chemotherapy—have shown limited efficacy in enhancing long-term survival and are frequently associated with substantial side effects [1–3]. These conventional approaches have not yielded substantial improvements in patient outcomes, necessitating the exploration of novel diagnostic and therapeutic strategies [1–4].

Recent studies have emphasized non-invasive diagnostic methods, particularly the potential of salivary biomarkers such as interleukins and tumor suppressor protein p53, for early detection and monitoring of OSCC [4]. Immunotherapy has also emerged as a promising treatment, especially for recurrent or metastatic OSCC, with checkpoint inhibitors proving effective in enhancing the immune response against cancer cells [5, 6]. Genomic analyses in clinical trials have identified key genetic mutations, including those in the CUL3 and ZFP36L2 genes, offering new pathways for personalized treatment strategies [7, 8]. These advancements highlight the importance of early diagnosis and targeted therapies in improving outcomes for OSCC patients.

The Gαi (inhibitory heterotrimeric G proteins) family consists of three subunits, Gαi1, Gαi2, and Gαi3 [9, 10]. These proteins are traditionally associated with G protein-coupled receptors, where they function to inhibit adenylate cyclase, leading to reduced intracellular cyclic AMP levels [1]. Recent studies have established that Gαi1 and Gαi3 are crucial for transmitting signals from a variety of receptor tyrosine kinases (RTKs), including epidermal growth factor receptor (EGFR), fibroblast growth factor receptor, keratinocyte growth factor receptor, stem cell factor receptor (c-Kit), brain-derived neurotrophic factor receptor (TrkB), and vascular endothelial growth factor receptor 2 [11–16]. These Gαi proteins play essential roles in mediating oncogenic signaling pathways, such as Akt-mTORC1 [11–16].

In addition to RTKs, Gαi1 and Gαi3 have been shown to interact with various non-RTK receptors, including the leucine-rich repeat-containing G-protein coupled receptor 4 (LGR4) for R-spondin 3 (RSPO3), the CD146 for Netrin-1, the receptor for interleukin-4 and lipopolysaccharide receptor Toll-Like Receptor 4 [17–20]. Through these interactions, Gαi proteins facilitate the activation of the Akt-mTOR axis, underscoring their significance as oncogenic drivers and potential therapeutic targets in cancer. Indeed, recent investigations have revealed that Gαi proteins are frequently overexpressed in a range of human cancers, highlighting their pivotal roles in tumor initiation and progression [21–23]. This research aims to elucidate the expression patterns and functional significance of Gαi3 in OSCC, providing deeper insights into its potential as a biomarker and target for therapeutic intervention.

## Results

### Gαi3 is upregulated in human OSCC tissues and cells

**Fig. 1:**
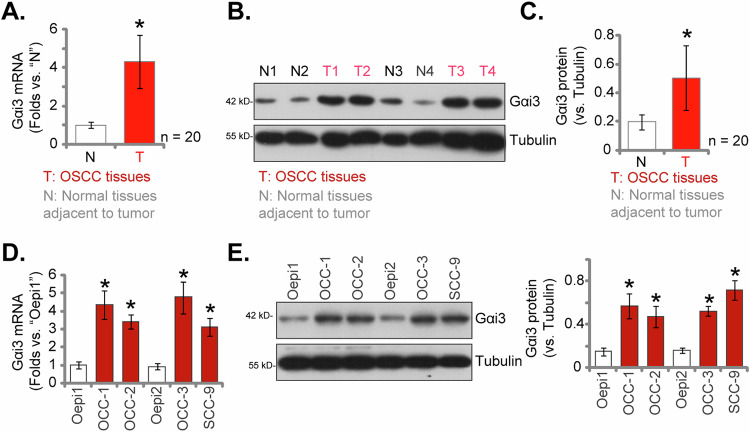
Gαi3 is upregulated in human OSCC tissues and cells. The expression of *Gαi3* mRNA and protein was assessed in twenty pairs (*n* = 20) of OSCC tissues (“T”) and adjacent normal tissues (“N”) through qPCR (**A**) and western blotting (**B**, **C**) assays. The expression of *Gαi3* mRNA and protein in a panel of primary and established human OSCC cells, as well as primary human oral cavity epithelial cells (“Oepi1-2”), was evaluated using qPCR (**D**) and western blotting (**E**) assays, respectively. Results were presented as mean values ± standard deviation (SD). *P < 0.05 compared to “N” tissues (**A**–**C**) or “Oepi1” cells (**D**, **E**). Scale bar = 100 μm.

Firstly, we examined the expression of Gαi3 in local OSCC tissues. Samples were collected from OSCC patients, encompassing both tumor tissues and adjacent normal tissues from twenty distinct individuals. Quantitative PCR (qPCR) analysis, in Fig. 1A, revealed a striking increase in *Gαi3* mRNA levels within OSCC tissues (“T”) compared to adjacent normal tissues (“N”). Western blotting assays were then employed to assess Gαi3 protein expression, which demonstrated a significant elevation in OSCC tissues across two representative patients (Patient #1 and Patient #2, Fig. 1B). Pooling the blotting data from all twenty tissue sets, we observed a substantial upregulation of Gαi3 protein in OSCC tissues (Fig. 1C).

The expression of Gαi3 was evaluated in various human OSCC cells. Primary OSCC cells derived from three patients, designated as OCC-1, OCC-2, and OCC-3 (reported in our previous studies [24, 25]), along with the immortalized cell line SCC-9, were cultured for analysis. Results from the qPCR assay revealed a significant increase in *Gαi3* mRNA expression in both the primary and established OSCC cells, as compared to that in primary human oral cavity epithelial cells (“Oepi1-2”, [24, 25]) (Fig. 1D). Moreover, elevated levels of Gαi3 protein were observed in both primary and established OSCC cells (Fig. 1E). Collectively, these findings provide compelling evidence of Gαi3 upregulation in OSCC tissues and cells.

### Silencing of Gαi3 leads to significant anti-cancer effects in OSCC cells

**Fig. 2:**
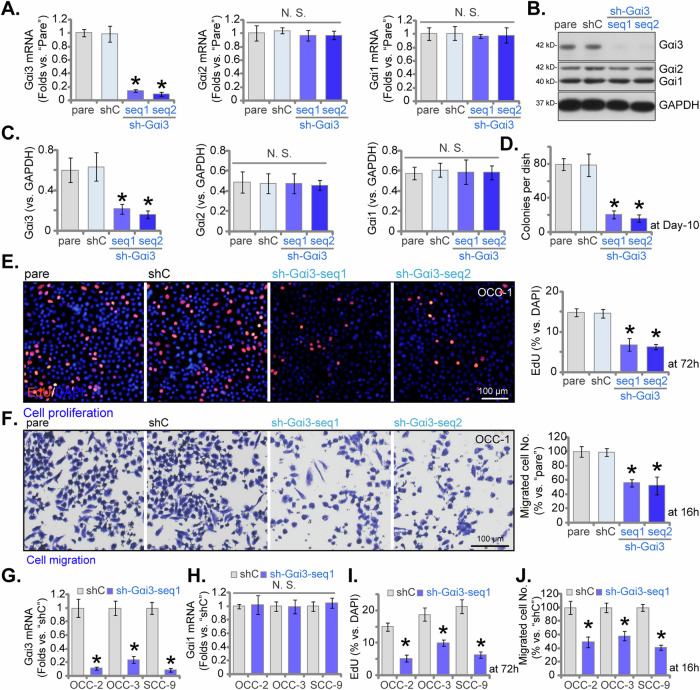
Silencing of Gαi3 leads to significant anti-cancer effects in OSCC cells. Primary and established human OSCC cells underwent stable transduction with Gαi3 lentivirus shRNA (sh-Gαi3-seq1/sh-Gαi3-seq2, two distinct sequences) or a scramble control shRNA (“shC”). Subsequently, the expression levels of the listed mRNAs and proteins were evaluated using qPCR (**A,** **G**, **H**) and western blotting (**B**, **C**) assays, respectively. Following culturing for specified durations, assays were conducted to assess colony formation (**D**), cell proliferation (quantification of EdU-positive nuclei ratio, **E**, **I**), cell migration (“Transwell” assays, **F**, **J**). The designation “pare” denotes parental control cells. Data were expressed as mean ± standard deviation (SD, *n* = 5). Statistical significance was denoted by *P < 0.05 compared to the “shC” group, while “N .S.” indicated non-statistically significant differences (*P* > 0.05). Experiments were repeated five times with consistent results. Scale bar = 100 μm.

To explore the potential function role of Gαi3 in OSCC cells, we employed a shRNA-mediated approach to silence Gαi3. Specifically, the primary human OSCC cells OCC-1 [24, 25] were transfected individually with two distinct lentiviral short hairpin RNAs (shRNAs) targeting Gαi3, namely sh-Gαi3-seq1 and sh-Gαi3-seq2 [22, 26]. Following selection using puromycin, stable cells were established. Subsequent qPCR assays revealed a remarkable reduction of over 90% in *Gαi3* mRNA expression in OCC-1 cells expressing sh-Gαi3-seq1/2 compared to controls with scramble control shRNA (“shC”) (Fig. 2A). Notably, the mRNA expression of *Gαi1* and *Gαi2* remained unaffected (Fig. 2A). Confirmation of Gαi3 protein silencing in stable OCC-1 cells was obtained through western blotting assays, as depicted in Fig. 2B, C. Consistently, the protein expression of Gαi1 and Gαi2 remained unaltered (Fig. 2B, C).

The OCC-1 cell colony formation was largely inhibited after Gαi3 silencing (Fig. 2D). Figure 2E illustrated a potent inhibition of OCC-1 cell proliferation by Gαi3 shRNA, evidenced by a significant decrease in the ratio of EdU-stained nuclei in OCC-1 cells expressing sh-Gαi3-Seq1/2 (Fig. 2E). Moreover, the “Transwell” assay results demonstrated that shRNA-caused stable silencing of Gαi3 robustly suppressed in vitro migration (Fig. 2F) of OCC-1 cells. As expected, the scramble control shRNA, shC, exhibited no significant impact on Gαi1/2/3 expression (Fig. 2A–C) or the functional attributes of OSCC cells (Fig. 2D–F).

Experiments were then conducted to assess whether Gαi3 silencing could induce similar effects in other OSCC cells. Primary human OSCC cells derived from two additional OSCC patients, OCC-2 and OCC-3, along with the immortalized OSCC cell line, SCC-9, were subjected to stable transduction with lentiviral Gαi3 shRNA (sh-Gαi3-seq1). Results demonstrated robust downregulation of *Gαi3* mRNA in both primary and immortalized OSCC cells (Fig. 2G), with no impact on *Gαi1* mRNA expression (Fig. 2H). Notably, Gαi3 shRNA exhibited potent inhibition of cell proliferation, as evidenced by the reduced EdU-positive nuclei ratio (Fig. 2I), and migration, as demonstrated by “Transwell” assays (Fig. 2J), in primary OSCC cells and established SCC-9 cells.

### Silencing of Gαi3 leads to apoptosis in OSCC cells

**Fig. 3:**
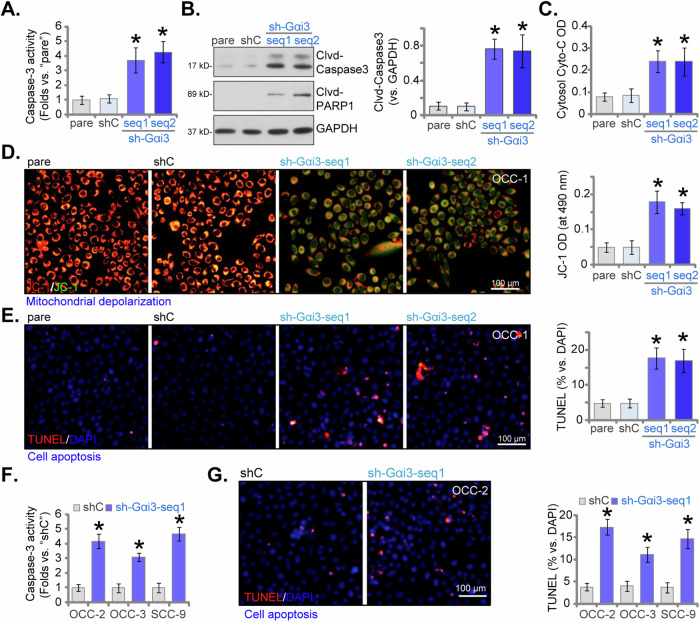
Silencing of Gαi3 leads to apoptosis in OSCC cells. Primary and established human OSCC cells underwent stable transduction with Gαi3 lentivirus shRNA (sh-Gαi3-seq1/sh-Gαi3-seq2, two distinct sequences) or a scramble control shRNA (“shC”). Following culturing for 96 h, the caspase-3 activity (**A**, **F**), expression of apoptosis-related proteins (**B**), the cytosol cytochrome C content (ELISA assay, **C**), and mitochondrial depolarization (JC-1 staining assay, **D**) were tested; Cell apoptosis was measured via nuclear TUNEL staining (**E**, **G**) assays. Data were expressed as mean ± standard deviation (SD, *n* = 5). Statistical significance was denoted by *P < 0.05 compared to the “shC” group. Experiments were repeated five times with consistent results. Scale bar = 100 μm.

Given that Gαi3 shRNA led to growth arrest and inhibition of proliferation in both primary and established OSCC cells, we proceeded to investigate its potential impact on cell apoptosis. Notably, the activity of caspase-3 was significantly elevated in stable OCC-1 cells expressing sh-Gαi3-seq1/2 (Fig. 3A). Western blotting analysis further revealed that Gαi3 shRNA triggered cleavages of caspase-3 and poly (ADP-ribose) polymerase 1 (PARP1) in OCC-1 cells (Fig. 3B), supporting apoptosis induction. Consistent with this, we observed a substantial increase in cytosolic cytochrome C levels in Gαi3-silenced OCC-1 cells (Fig. 3C). Additionally, the accumulation of JC-1 green monomers suggested mitochondrial depolarization in Gαi3-silenced OCC-1 cells (Fig. 3D), further supporting apoptosis. This was corroborated by an increase in TUNEL-positive nuclei in OCC-1 cells upon sh-Gαi3-seq1/2 treatment (Fig. 3E). In contrast, the scramble control shRNA, shC, failed to induce apoptosis activation in OCC-1 cells (Fig. 3A–E).

In other primary OSCC cells, OCC-2/OCC-3, and the immortalized cell line SCC-9, silencing of Gαi3 using sh-Gαi3-seq1 (refer to Fig. 2) yielded comparable outcomes. There was a significant increase in caspase-3 activity (Fig. 3F) observed after Gαi3 silencing. Furthermore, significant activation of apoptosis was evident in these OSCC cells expressing sh-Gαi3-seq1, as indicated by markedly elevated TUNEL-positive nuclei staining (Fig. 3G).

### Knocking out of Gαi3 induces significant anti-cancer effects in OSCC cells

**Fig. 4:**
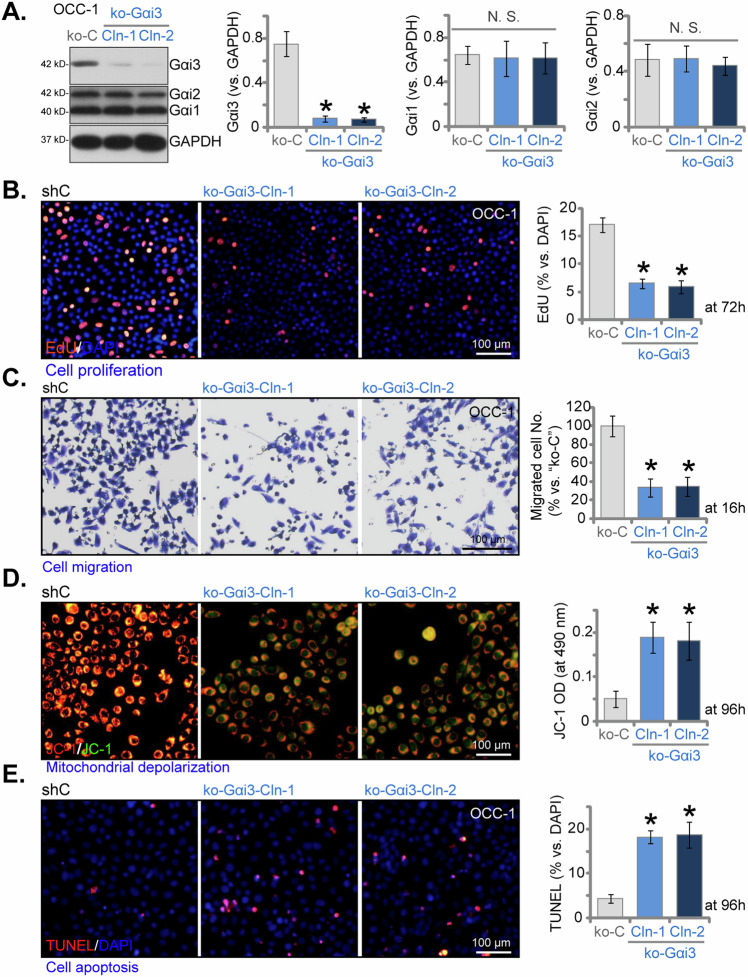
Knocking out of Gαi3 induces significant anti-cancer effects in OSCC cells. Cas9-expressing OCC-1 primary cells were utilized to establish two stable colonies, namely “ko-Gαi3-Cln-1 and ko-Gαi3-Cln-2”, harboring the CRISPR/Cas9-Gαi3-KO-puro construct, along with a control group transfected with the CRISPR/Cas9 empty vector (“koC”). The expression levels of the listed proteins were evaluated via western blotting assays (**A**). Subsequently, cells were cultured for specified durations, and assays were conducted to evaluate cell proliferation (quantification of EdU-positive nuclei ratio, **B**), cell migration (“Transwell” assays, **C**), mitochondrial depolarization (JC-1 staining assay, **D**), and apoptosis (measuring TUNEL-positive nuclei ratio, **E**). Data were expressed as mean ± standard deviation (SD, *n* = 5). Statistical significance was denoted by *P < 0.05 compared to the “koC” group, while “N. S.” indicated non-statistically significant differences (*P* > 0.05). Experiments were repeated five times with consistent results. Scale bar = 100 μm.

To further reinforce the pivotal role of Gαi3 in OSCC cells, we employed the CRISPR/Cas9 strategy to completely knockout (KO) Gαi3. Following transduction of the Cas9-expressing OCC-1 primary cells with the CRISPR/Cas9-Gαi3-KO-puro construct, we successfully established two stable clones, ko-Gαi3-Cln-1 and ko-Gαi3-Cln-2, subsequent to screening for Gαi3 KO (Fig. 4A). As shown, Gαi3 protein expression was effectively depleted in the ko-Gαi3-Cln-1/2 OCC-1 cells (Fig. 4A), while the expression of Gαi1 and Gαi2 proteins remained intact (Fig. 4A). The inhibition of cell proliferation in OCC-1 cells following CRISPR/Cas9-induced Gαi3 KO was evident, as evidenced by reduced nuclear EdU staining (Fig. 4B). Furthermore, the results from the “Transwell” assay demonstrated hindered in vitro migration (Fig. 4C) of OCC-1 cells post Gαi3 KO. The accumulation of JC-1 green monomers further supported mitochondrial depolarization in ko-Gαi3-Cln-1/2 OCC-1 cells (Fig. 4D). Additionally, apoptosis activation was detected in ko-Gαi3 OCC-1 cells, with a significantly increased TUNEL-positive nuclei ratio (Fig. 4E). Overall, these findings further highlighted the crucial role of Gαi3 in OSCC cell progression.

### Ectopic overexpression of Gαi3 fosters the growth of OSCC cells

**Fig. 5:**
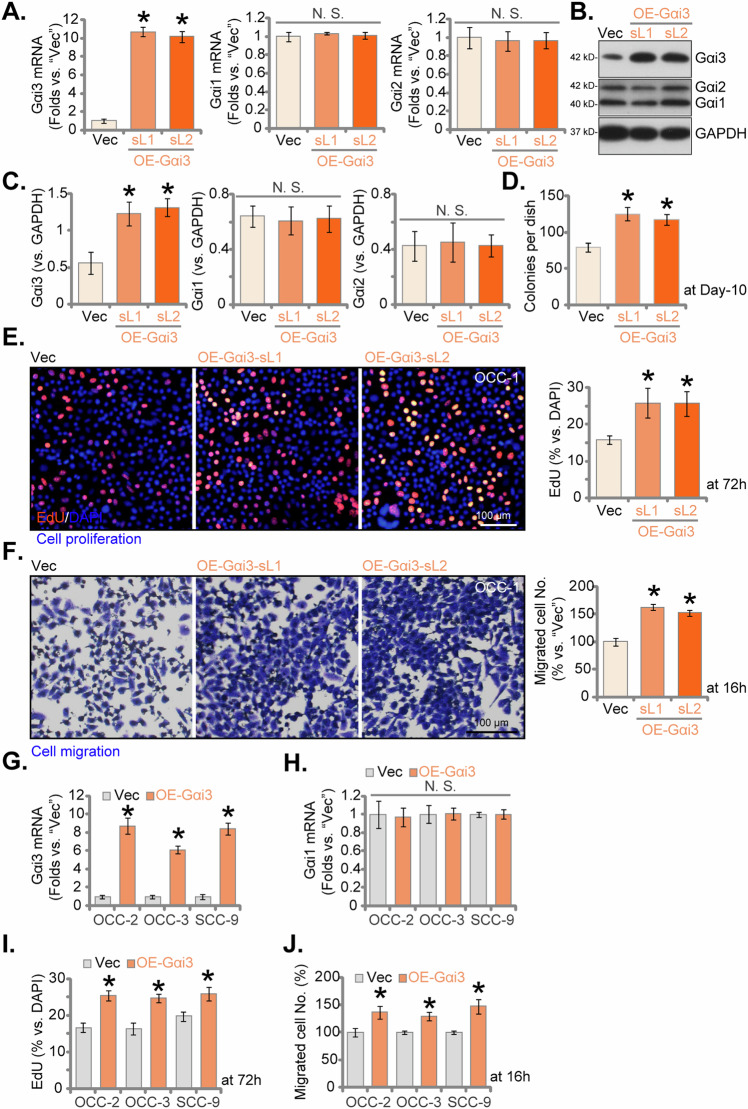
Ectopic overexpression of Gαi3 fosters the growth of OSCC cells. Primary human OSCC cells (OCC-1, OCC-2, OCC-3) and immortalized OS cell line (SCC-9) were stably transduced with a lentiviral construct containing either wild-type Gαi3 (“OE-Gαi3”) or an empty vector (“Vec”). Subsequently, the expression levels of specific mRNAs and proteins were evaluated through qPCR and western blotting assays (**A**–**C**, **G**, **H**). The cells were cultured for designated time intervals. Various functional assays were performed: colony formation (**D**), cell proliferation (assessed by the ratio of EdU-positive nuclei, **E**, **I**), and migration (via “Transwell” assays, **F**, **J**). Data were expressed as mean ± standard deviation (SD, *n* = 5). Statistical significance was denoted by *P < 0.05 compared to the “Vec” group, while “N. S.” indicated non-statistically significant differences (*P* > 0.05). Experiments were repeated five times with consistent results. Scale bar = 100 μm.

Subsequently, we proceeded by introducing a lentiviral construct harboring *Gαi3* cDNA, as reported in prior investigations, into OCC-1 primary cells. Following a selection process, we successfully established two distinct populations of OCC-1 cells with overexpressed Gαi3, designated as “OE-Gαi3-sL1” and “OE-Gαi3-sL2”. Comparative analysis with control cells transfected with an empty vector (“Vec”) revealed a remarkable increase in both *Gαi3* mRNA expression (Fig. 5A) and protein levels (Fig. 5B, C) within the OE-Gαi3 OCC-1 cells. Functionally, our investigations unveiled the profound impact of ectopic Gαi3 overexpression on various cellular processes in OCC-1 cells. Importantly, heightened Gαi3 expression significantly enhanced colony formation capabilities (Fig. 5D). Furthermore, it accelerated cell proliferation, as evidenced by a significant increase in the ratio of EdU-positive nuclei (Fig. 5E). Additionally, our findings from “Transwell” assays, Fig. 5F, underscored a pronounced enhancement in cell migration facilitated by Gαi3 overexpression.

Furthermore, in other primary OSCC cells, OCC-2 and OCC-3, alongside the immortalized cell line, SCC-9, the utilization of the identical construct (“OE-Gαi3”) elicited significant increase in *Gαi3* mRNA expression (Fig. 5G), while no significant impact on *Gαi1* mRNA levels was observed (Fig. 5H). Notably, the introduction of OE-Gαi3 facilitated a marked augmentation in cell proliferation, as evidenced by the heightened ratio of EdU-positive nuclei (Fig. 5I), and also enhanced migration capabilities (Fig. 5J). These findings underscore the consistent and robust influence of Gαi3 overexpression across different OSCC cells, further emphasizing its integral role in modulating key cellular processes critical for tumorigenesis and progression.

### Gαi3 plays a critical role in activating Akt-mTOR signaling in OSCC cells

**Fig. 6:**
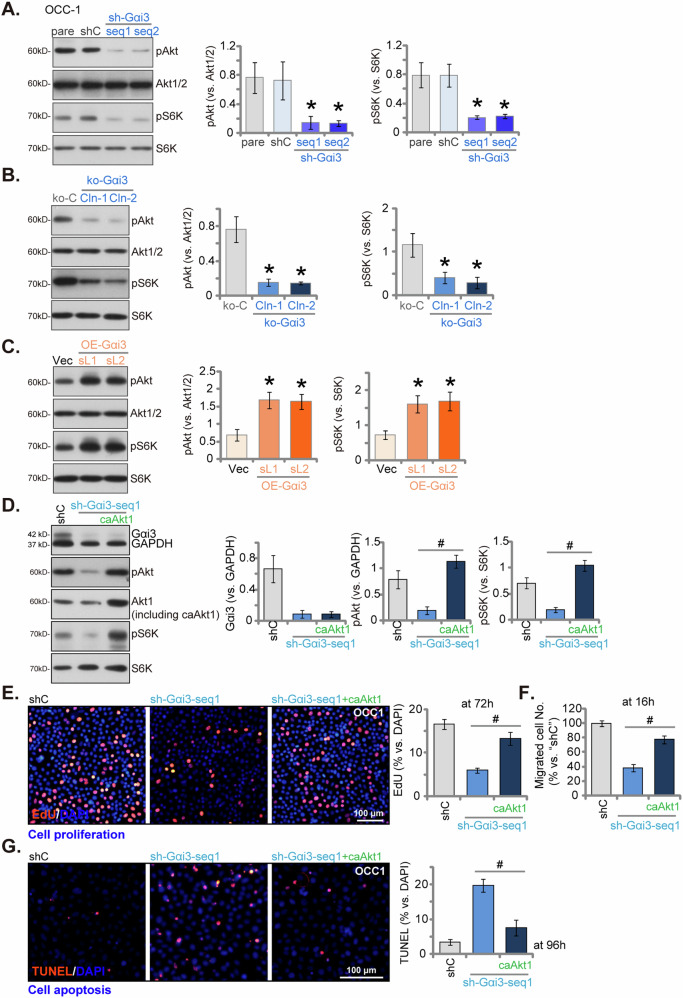
Gαi3 plays a critical role in activating Akt-mTOR signaling in OSCC cells. The OCC-1 primary cells underwent stable transduction with Gαi3 lentivirus shRNA (sh-Gαi3-seq1/sh-Gαi3-seq2, two distinct sequences), a scramble control shRNA (“shC”), Cas9 plus CRISPR/Cas9-Gαi3-KO-puro construct (“ko-Gαi3-Cln-1 and ko-Gαi3-Cln-2”, two stable colonies), the CRISPR/Cas9 empty vector (“koC”), a lentiviral construct containing either wild-type Gαi3 (“OE-Gαi3”) or an empty vector (“Vec”), and expression of listed proteins was shown (**A**–**C**). OCC-1 primary cells transduced with Gαi3 lentivirus shRNA (sh-Gαi3-seq1) were further stably transduced with a constitutively active mutant Akt1 (caAkt1). The expression of the listed proteins was confirmed (**D**). Cells were then cultivated for the indicated time periods, and assays were performed to test cell proliferation, migration, and apoptosis via nuclear EdU staining (**E**), “Transwell” (**F**), and nuclear TUNEL staining (**G**), respectively. Data were expressed as mean ± standard deviation (SD, *n* = 5). Statistical significance was denoted by *P < 0.05 compared to the control genetic treatment group (**A**–**C**). ^**#**^ *P* < 0.05 (**D**–**G**). Experiments were repeated five times with consistent results. Scale bar = 100 μm.

Prior studies have confirmed Gαi3’s association with both RTKs [11, 13–15, 19, 23] and non-RTK receptors [17–19], underscoring its function in promoting the activation of the downstream Akt-mTOR signaling pathway. In our investigation into Akt-mTOR signaling, we observed a significant reduction in phosphorylated-Akt (Ser-473) and phosphorylated-S6K (Thr-389) levels in OCC-1 cells expressing shRNAs targeting Gαi3 (sh-Gαi3-seq1 and sh-Gαi3-seq2) (Fig. 6A). Similarly, in Gαi3 KO OCC-1 cells (ko-Gαi3-Cln-1 and ko-Gαi3-Cln-2), Akt-S6K phosphorylation was largely decreased (Fig. 6B). Conversely, ectopic overexpression of Gαi3 led to increased Akt-S6K phosphorylation in OCC-1 cells (Fig. 6C). To substantiate that Akt-mTOR inhibition was the primary mechanism underlying the anti-OSCC cell activity induced by Gαi3 depletion, we introduced a lentiviral constitutively active Akt1 (“caAkt1”) into sh-Gαi3-seq1-expressing OCC-1 cells, which rescued Akt and S6K phosphorylation (Fig. 6D). Importantly, caAkt1 alleviated proliferation inhibition (EdU assays, Fig. 6E), migration suppression (“Transwell” assays, Fig. 6F), and apoptosis induction (TUNEL assays, Fig. 6G) in sh-Gαi3-seq1 OCC-1 cells.

### Gαi3 silencing hinders subcutaneous OSCC xenograft growth in nude mice

**Fig. 7:**
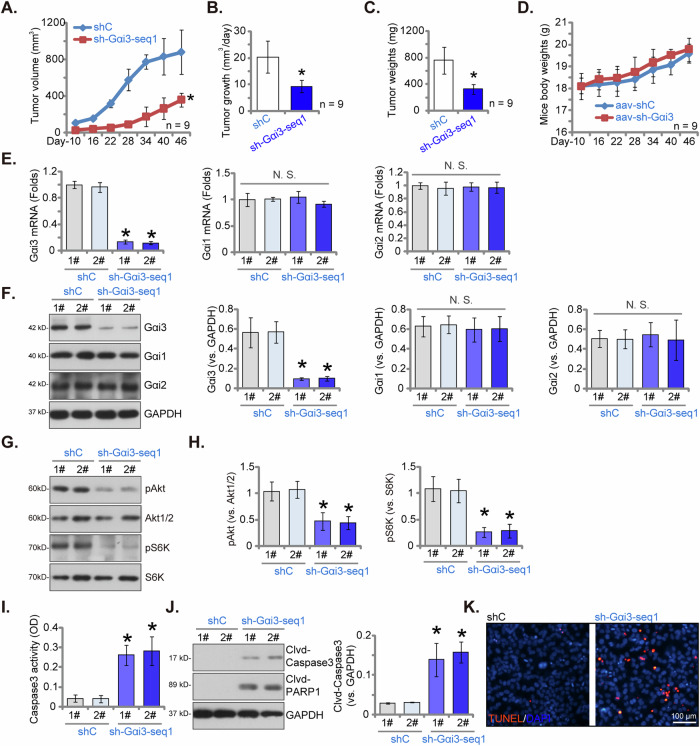
Gαi3 silencing hinders subcutaneous OSCC xenograft growth in nude mice. The stable OCC-1 cells expressing Gαi3 shRNA (“sh-Gαi3-seq1”) or control shRNA (“shC”) were subcutaneously injected into the flanks of nude mice at eight million cells per mouse, with nine mice per experimental group (*n* = 9). Recordings commenced ten days post-cell inoculation (day-10). The volumes of the OCC-1 xenografts (**A**) and the body weights of the mice (**D**) were monitored and recorded at six-day intervals from day-10 to day-46. The daily growth rate of the OCC-1 xenografts was also evaluated (**B**). On day-46, the xenografts were surgically excised, and their weights were measured (**C**). Two xenografts per group (labeled as “1#” and “2#”) were isolated, protein and mRNA expression analyses were performed on tissue lysates from the harvested xenografts (**E**–**H**, **J**), and Caspase-3 activity was also assessed (**I**). Additionally, xenograft sections underwent TUNEL/DAPI fluorescence staining (**K**). Data were expressed as mean ± standard deviation (SD). Statistical significance was denoted by *P < 0.05 compared to the “shC” group, while “N. S.” indicated non-statistically significant differences (*P* > 0.05). **A**–**D** *n* = 9 mice per group. **E**–**K**, each xenograft was cut into five pieces and tested separately. Scale bar = 100 μm.

To substantiate the critical role of Gαi3 in the in vivo growth of OSCC cells, we conducted an experiment utilizing OCC-1 cells. We subcutaneously injected eight million sh-Gαi3-seq1-expressing OCC-1 cells or shC-expressing OCC-1 cells into the flanks of nude mice. Recordings were initiated ten days post-cell inoculation. The tumor growth curve results revealed a marked reduction in the growth of sh-Gαi3-seq1 OCC-1 xenografts compared to shC OCC-1 xenografts (Fig. 7A). Daily growth rate quantification further corroborated the robust suppression of tumor growth in the sh-Gαi3-seq1 OCC-1 xenografts (Fig. 7B). Additionally, the sh-Gαi3-seq1 OCC-1 xenografts exhibited significantly lower weights compared to the Cas9-C OCC-1 xenografts (Fig. 7C), while the body weights of the mice remained indifferent (Fig. 7D). A substantial decrease in *Gαi3* mRNA and protein expression was observed in the sh-Gαi3-seq1 OCC-1 xenografts, whereas the expression levels of Gαi1 and Gαi2 remained unchanged (Fig. 7E, F).

Further investigation revealed a marked reduction in the phosphorylation levels of Akt and S6K1 in sh-Gαi3-seq1 OCC-1 xenograft tissues (Fig. 7G, H). Conversely, Caspase-3 activity, as well as the levels of cleaved-Caspase-3 and cleaved-PARP1, were significantly increased in the sh-Gαi3-seq1-expressing OCC-1 xenograft tissues (Fig. 7I, J). Further supporting the activation of apoptosis, tissue fluorescence assays showed a higher ratio of TUNEL-positive nuclei in the sh-Gαi3-seq1 OCC-1 xenograft tissue sections (Fig. 7K). These results collectively underscore that Gαi3 silencing significantly inhibited the growth of OCC-1 xenografts in nude mice, and inactivating the Akt-mTOR pathway and triggering apoptosis.

## Discussion

Recent advancements in targeted therapies for OSCC have yield promising results, especially in immunotherapy and molecular-targeted treatments [27–29]. Checkpoint inhibitors like pembrolizumab and nivolumab have improved survival in patients with recurrent or metastatic OSCC [27–29]. Genomic analyses have identified key mutations in *TP53*, *PIK3CA*, and *NOTCH1*, paving the way for personalized treatments. EGFR inhibitors and Akt-mTOR pathway inhibitors have shown potential in clinical trials as well [27–29]. Additionally, targeting the tumor microenvironment and exploiting synthetic lethality mechanisms are being explored to enhance efficacy and overcome resistance [27–29]. These developments underscore the importance of integrating molecular profiling into OSCC treatment.

Recent investigations have highlighted the pivotal role of Gαi proteins, specifically Gαi1 and Gαi3, in signal transduction mechanisms associated with various RTKs [11–15] and non-RTK receptors [17–20]. These proteins not only facilitate oncogenic pathways initiated by activated cell surface receptors but are also overexpressed in various malignancies, such as glioma, osteosarcoma, cervical cancer, and nasopharyngeal carcinoma, where their presence correlates with poor prognosis [12, 21–23, 26]. Notably, silencing or KO of Gαi3 has been shown to impede the growth of osteosarcoma, cervical cancer, and glioma cells, while its overexpression has been linked to enhanced cancer cell proliferation [12, 21–23, 26].

In the present study, we further investigated the role of Gαi3 as a novel therapeutic target in OSCC. Our experimental results demonstrated a significant upregulation of Gαi3 in OSCC tissues and cells compared to adjacent non-cancerous tissues and normal oral epithelial cells. Remarkably, the depletion of Gαi3 via shRNA or CRISPR-Cas9 gene editing resulted in robust anticancer effects in various OSCC cells and significantly inhibited the proliferation and migration of primary and established OSCC cells. Moreover, Gαi3 silencing or KO also resulted in significant apoptosis activation in OSCC cells. Conversely, the ectopic overexpression of Gαi3 was found to accelerate OSCC cell proliferation and migration, reinforcing the potential of Gαi3 as a promising target for OSCC therapy. In vivo, Gαi3 silencing significantly suppressed the growth of subcutaneous OSCC xenografts in nude mice.

The Akt-mTOR signaling pathway plays a pivotal role in the pathogenesis of OSCC [30, 31]. Overactivation of Akt fosters cellular proliferation and survival by phosphorylating and inhibiting pro-apoptotic factors, thereby enhancing resistance to apoptosis [30, 31]. Additionally, Akt regulates cell cycle progression by modulating key regulatory proteins, stimulates glucose metabolism to meet the energy demands of proliferating cells, and activates transcription factors that drive the expression of pro-survival and proliferative genes [30, 31]. Downstream, mTOR regulates protein synthesis and cellular metabolism, contributing to tumor growth and progression. Moreover, Akt-mTOR signaling facilitates angiogenesis, ensuring a sustained supply of nutrients and oxygen to the tumor [30, 31]. Given its integral role in OSCC development, the Akt-mTOR pathway represents a compelling target for therapeutic intervention.

Previous studies have extensively documented Gαi3’s interaction with various RTKs [11, 13–15, 19, 23] and non-RTK receptors [17–19], underscoring its significant role in the activation of the Akt-mTOR signaling cascade. In this study, we have demonstrated that Gαi3 plays a crucial role in the activation of the Akt-mTOR signaling pathway in OSCC cells. Specifically, silencing or KO of Gαi3 resulted in a significant decrease in the phosphorylation levels of both Akt and S6K, key components of this pathway. Conversely, overexpression of Gαi3 led to an increase in their phosphorylation levels. Moreover, the restoration of Akt-mTOR activation through the introduction of caAkt1 was able to mitigate the anti-OSCC effects that were induced by Gαi3 shRNA. Additionally, we observed that Akt-mTOR activation was notably reduced in OSCC xenografts where Gαi3 had been silenced. This underscores the importance of Gαi3 in maintaining the activity of the Akt-mTOR pathway in OSCC.

## Materials and methods

### Chemicals and reagents

Puromycin, cell culture medium, serum, polybrene, and RNA assay reagents were obtained from Sigma-Aldrich (St. Louis, MO). Fluorescent dyes, including EdU (5-ethynyl-2′-deoxyuridine), TUNEL (terminal deoxynucleotidyl transferase dUTP nick end labeling), DAPI, and JC-1, were sourced from Biyuntian (Wuxi, China). Antibodies, mRNA primers, and viral constructs utilized in this study were kindly provided by Dr. Cao at Soochow University.

### Cells and human tissues

As reported in our previous studies [24, 25], four patients with oral cavity carcinoma (OCC) provided written-informed consent to participate in this study. During surgery, OCC tissues and adjacent normal epithelial tissues were carefully separated. The tissues were washed and digested with 0.1% collagenase I, and the resulting cell suspensions were filtered through a 70 μm nylon cell strainer. Primary cells were cultured in complete DMEM/F12 medium (with FBS) supplemented with basic fibroblast growth factor and epidermal growth factor. This process resulted in the establishment of four different primary OCC cells (“OCC-1/2/3/4”) and two oral cavity epithelial cell lines (“Oepi1-2”). The established SCC-9 cells were maintained in DMEM medium plus 10% fetal bovine serum (FBS) [24, 25]. Fresh OSCC tissues and adjacent normal oral epithelial tissues were collected from twenty primary OSCC patients at the Second Affiliated Hospital of Soochow University. Tissues were stored in liquid nitrogen. Written-informed consent was obtained from each patient. All procedures involving human tissues/cells were approved by the Institutional Ethics Committee and Internal Review Committee of Soochow University and conducted in accordance with the Declaration of Helsinki.

### Western blotting

Following the extraction of total proteins, quantification was conducted utilizing the bicinchoninic acid protein assay kit (Thermo Fisher Scientific, Waltham, MA). Equal quantities of protein samples were subjected to separation by 10–12% sodium dodecyl sulfate-polyacrylamide gel electrophoresis. The resolved proteins were then transferred onto polyvinylidene fluoride membranes at 4 °C. The membranes were incubated sequentially with 5% non-fat milk-block buffer, primary and secondary antibodies at 4 °C. Detection of protein signals was performed using the Amersham ECL Plus Western Blotting Detection System. Detailed images of the uncropped blots are provided in Fig. S1.

### RNA isolation and qPCR

Total RNA was isolated from cell and tissue lysates using TRIzol reagent (Biyuntian, Wuxi, China). The concentration and purity of the RNA samples were evaluated with a Nanodrop 2000 spectrophotometer. Complementary DNA (cDNA) synthesis was carried out using the Promega M-MLV Reverse Transcriptase kit. qPCR was subsequently performed with the SYBR Green PCR Master Mix (Thermo Fisher Scientific, Waltham, MA). Relative quantification of gene expression was calculated using the 2^−ΔΔ^Cq method, with *glyceraldehyde 3-phosphate dehydrogenase* serving as the internal control.

### shRNA-mediated silencing of Gαi3

Lentiviral vectors encoding two distinct shRNAs targeting human Gαi3 (GNAI3) (“sh-Gαi3-seq1” and “sh-Gαi3-seq2”) were generously provided by Dr. Cao from Soochow University [16, 22, 23, 26, 32], utilizing the GV369 construct. OSCC cells or epithelial cells were cultured in FBS-containing complete medium supplemented with polybrene to enhance transduction efficiency. The cells were then infected with the lentivirus at a multiplicity of infection (MOI) of 10.8 for 36 h. Post-infection, the cells were maintained in a medium containing puromycin for an additional ten days to ensure the selection of stable cells. The knockdown efficiency of Gαi3 expression in these stable cells was rigorously monitored and validated using qPCR and Western blotting analyses.

### Gαi3 overexpression

The overexpression of Gαi3 was achieved utilizing a lentiviral vector harboring the Gαi3-expressing sequence, generously provided by Dr. Cao [16, 18, 22, 23, 26]. OSCC cells were cultured in FBS-containing medium supplemented with polybrene to enhance viral transduction efficiency. The cells were subsequently infected with the Gαi3-expressing lentivirus at a MOI of 9.6 for a duration of 36 h. Post-infection, the cells were maintained in a medium containing puromycin for an additional nine days to establish stable cells overexpressing Gαi3. The verification of Gαi3 expression levels in the transduced cells was performed using qPCR and Western blotting analyses.

### Gαi3 knockout (KO)

To achieve Gαi3 KO, the sequence encoding the small-guide RNA targeting human Gαi3 (GNAI3) was integrated into the lenti-CRISPR/Cas9-KO-puro construct, also provided by Dr. Cao [16, 18, 22, 23, 26]. OSCC cells were engineered to stably expresses Cas9 and were then transfected with the lenti-CRISPR/Cas9-Gαi3-KO construct. Post-transfection, the cells were seeded into 96-well plates and cultured in medium containing puromycin to select for stable colonies. The expression levels of Gαi3 in these colonies were evaluated through Western blotting. The cell clone demonstrating the most substantial downregulation of Gαi3 protein was selected for subsequent experiments.

### Colony formation assay

OCC-1 cells (3 × 10^4^ per 10 cm dish) were trypsinized and resuspended in complete medium containing 0.5% agarose. Ten days after the specified treatments, surviving colonies were stained and manually counted.

### Nuclear TUNEL/EdU staining

Treated cancer cells or epithelial cells were seeded onto coverslips in 24-well plates and cultured for the designated durations. Cells were fixed with 4% formaldehyde-PBS for 8 min at room temperature, followed by permeabilization with 0.15% Triton X-100 in PBS for 5 min. Nuclei were stained using TUNEL, EdU, or DAPI dyes. Fluorescent images were captured using a Nikon fluorescence microscope.

### “Transwell” assays

“Transwell” chambers (Corning, NY, US) were used with 130 μL of cell suspension containing 8000 cells added to each chamber. The lower chamber was filled with 550 μL of medium containing 8% FBS. After 16 h of incubation, non-migrated cells were removed. Migrated cells were stained with 0.1% crystal violet in a solution containing 20% methanol for 12 min, washed with PBS, and photographed.

### Caspase-3 activity assessment

Caspase-3 activity was measured using a commercial kit from Biyuntian (Wuxi, China). Cells were seeded into 96-well plates at a density of 4000 cells per well and subjected to the specified treatments. Cell lysates were then incubated with a loading solution containing the caspase-3 substrate for 45 min. Caspase-3 activity was then determined using a fluorescence microplate reader (BioTek Synergy) with an emission wavelength of 620 nm.

### Cytochrome C detection

The Cytochrome C ELISA assay was performed using a commercial kit (Biyuntian, Wuxi, China) following the manufacturer’s protocol. The cytosol lysates were collected and were added to the attached 96-well plate. Non-specific sites were blocked using the attached blocking buffer. After washing, a primary antibody specific for cytochrome C was added, followed by incubation with an HRP-conjugated secondary antibody. The TMB substrate solution was then added to initiate the colorimetric reaction, which was subsequently terminated with a stop solution. Absorbance was measured at 450 nm using a microplate reader.

### Detection of mitochondrial depolarization (ΔΨm)

To assess mitochondrial membrane potential (MMP) reduction, the JC-1 dye assay (Invitrogen) was employed as previously described [24, 25]. After treatment, cells were incubated with JC-1 dye at a concentration of 4.0 μg/mL. Following staining, cells were thoroughly washed to remove excess dye. The green fluorescence intensity of JC-1, which correlates with a decrease in MMP (ΔΨm), was measured immediately captured by a fluorescence microscope, with its intensity recorded at 490 nm (indicating monomers intensity) [24, 25].

### Constitutively active mutant Akt1 (caAkt1)

Primary human OSCC cells were infected with the lentivirus bearing the constitutively active S473D mutant of Akt1 (“caAkt1”), as well as an empty vector-expressing virus, as described previously [22, 26, 33]. Stable cells expressing caAkt1 were established through selection using puromycin.

### Xenograft studies

Eight million OCC-1 cells per mouse were subcutaneously inoculated into the flanks of 5–6 week-old BALB/c nude mice (18–19 g, evenly divided by sex), obtained from the Animal Center of Shanghai Institutes for Biological Sciences (Shanghai, China). Xenograft tumors were established within 10 days post inoculation. Tumor volumes were measured every 6 days using a modified ellipsoid formula: (*π*/6) × *AB*^2^, where *A* is the longest axis and *B* is the shortest perpendicular axis of the tumor mass. Tumor tissue lysates were subjected to qPCR and Western blot assays. The sections were stained with TUNEL (Biyuntian, Wuxi, China). After washing, the sections are counterstained with DAPI to visualize nuclei. The sections are then mounted with an anti-fade medium and examined under a fluorescence microscope. The animal experiments were approved by the Institutional Animal Care and Use Committee and the Ethics Review Board of Soochow University.

### Statistical analysis

In vitro experiments were conducted with at least five biological replicates, and the results were reported as mean ± standard deviation. Statistical analyses were performed using GraphPad Prism software. Comparisons between two groups were made using the two-tailed Student’s *t*-test, while comparisons among more than two groups were analyzed using one-way ANOVA followed by Tukey’s post hoc test. A *P* value of less than 0.05 was considered statistically significant.

## Supplementary information

Original data

## Data Availability

All data generated during this study are included in this published article. Data will be made available upon request.
