# Supplementary figures and images for: The critical role of Gαi3 in oral squamous cell carcinoma cell growth
Source: Cell Death Discov. 2024 Oct 1;10:420. doi: 10.1038/s41420-024-02191-0 (PMC11443079; doi:10.1038/s41420-024-02191-0)

**Figure S1 the uncropped blotting images.**

**Figure 1.**

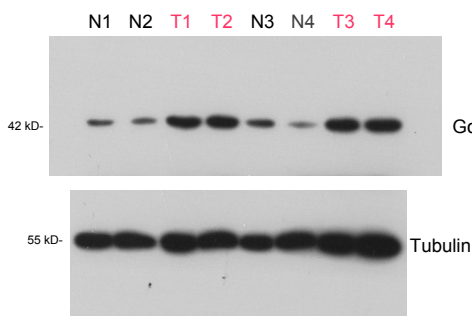

**Figure 2.**

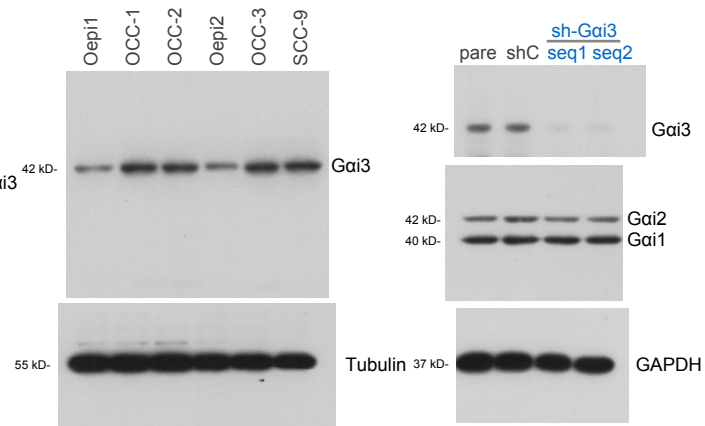

**Figure 3.**

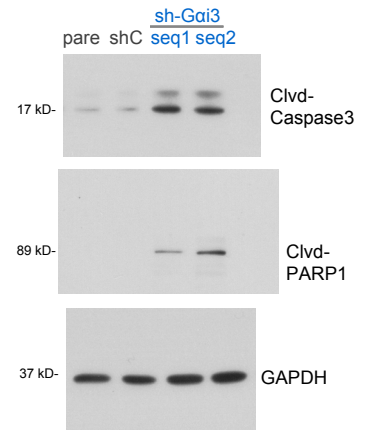

**Figure 4.**

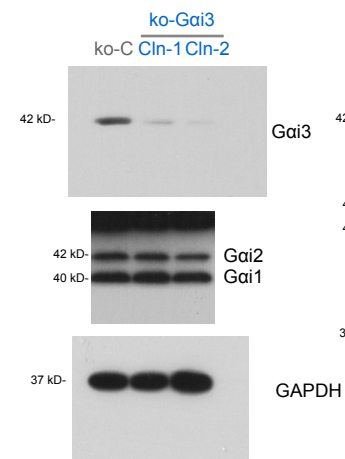

**Figure 5.**

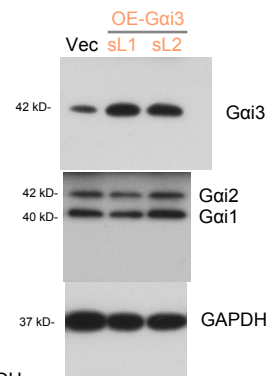

**Figure 7.**

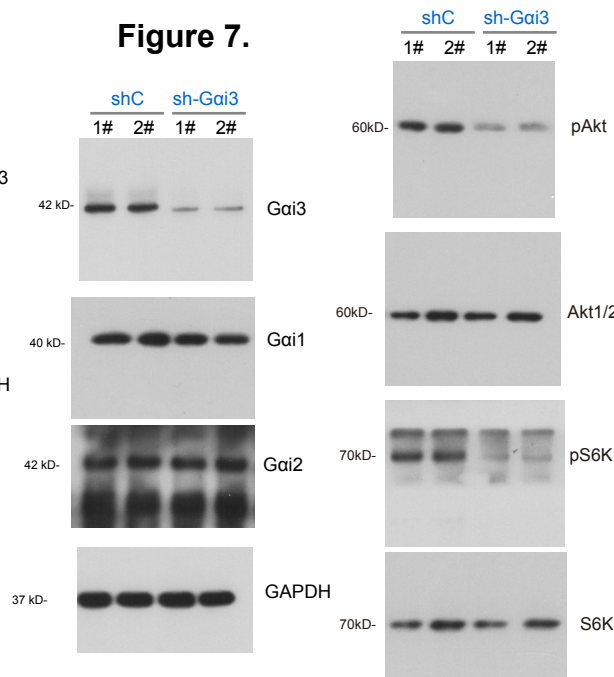

**Figure 6.**

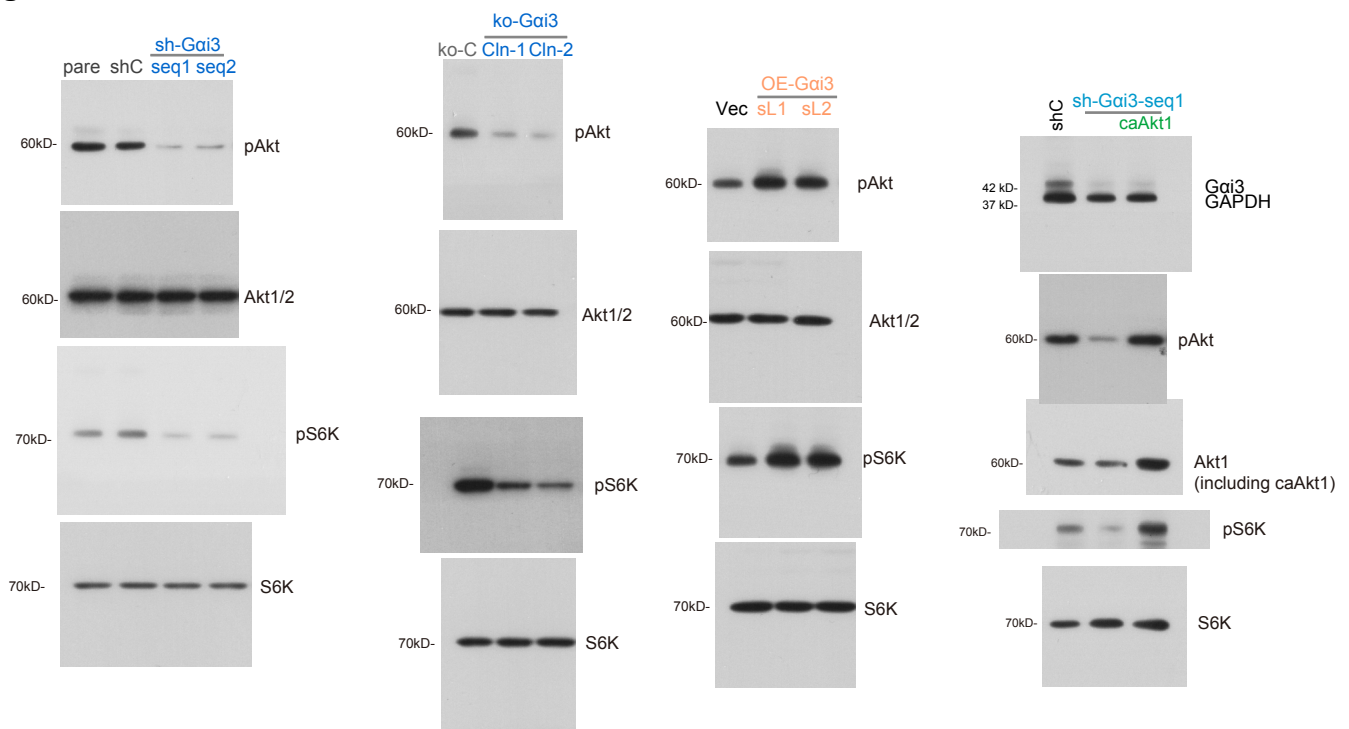

Supplement: Supplementary file 1 — Original data [file 41420_2024_2191_MOESM1_ESM.pdf]
